# Combining RNAscope and immunohistochemistry to visualize inflammatory gene products in neurons and microglia

**DOI:** 10.3389/fnmol.2023.1225847

**Published:** 2023-08-17

**Authors:** Jayson B. Ball, Connor J. McNulty, Suzanne M. Green-Fulgham, Joseph M. Dragavon, Igor R. Correia Rocha, Maggie R. Finch, Emily D. Prévost, Imaad I. Siddique, Brodie J. Woodall, Linda R. Watkins, Michael V. Baratta, David H. Root

**Affiliations:** ^1^Department of Psychology and Neuroscience, Center for Neuroscience, University of Colorado Boulder, Boulder, CO, United States; ^2^Advanced Light Microscopy Core, Biofrontiers Institute, University of Colorado Boulder, Boulder, CO, United States

**Keywords:** hybridization, spinal cord, neuroinflammation, NLRP3, interleukin-1beta, Imaris, rat, co-localization

## Abstract

A challenge for central nervous system (CNS) tissue analysis in neuroscience research has been the difficulty to codetect and colocalize gene and protein expression in the same tissue. Given the importance of identifying gene expression relative to proteins of interest, for example, cell-type specific markers, we aimed to develop a protocol to optimize their codetection. RNAscope fluorescent *in situ* hybridization (FISH) combined with immunohistochemistry (IHC) in fixed (CNS) tissue sections allows for reliable quantification of gene transcripts of interest within IHC-labeled cells. This paper describes a new method for simultaneous visualization of FISH and IHC in thicker (14-μm), fixed tissue samples, using spinal cord sections. This method’s effectiveness is shown by the cell-type-specific quantification of two genes, namely the proinflammatory cytokine interleukin-1beta (IL-1b) and the inflammasome NLR family pyrin domain containing 3 (NLRP3). These genes are challenging to measure accurately using immunohistochemistry (IHC) due to the nonspecificity of available antibodies and the hard-to-distinguish, dot-like visualizations of the labeled proteins within the tissue. These measurements were carried out in spinal cord sections after unilateral chronic constriction injury of the sciatic nerve to induce neuroinflammation in the spinal cord. RNAscope is used to label transcripts of genes of interest and IHC is used to label cell-type specific antigens (IBA1 for microglia, NeuN for neurons). This combination allowed for labeled RNA transcripts to be quantified within cell-type specific boundaries using confocal microscopy and standard image analysis methods. This method makes it easy to answer empirical questions that are intractable with standard IHC or *in situ* hybridization alone. The method, which has been optimized for spinal cord tissue and to minimize tissue preparation time and costs, is described in detail from tissue collection to image analysis. Further, the relative expression changes in inflammatory genes NLRP3 and IL-1b in spinal cord microglia vs. neurons of somatotopically relevant laminae are described for the first time.

## 1. Introduction

**Figure 1:**
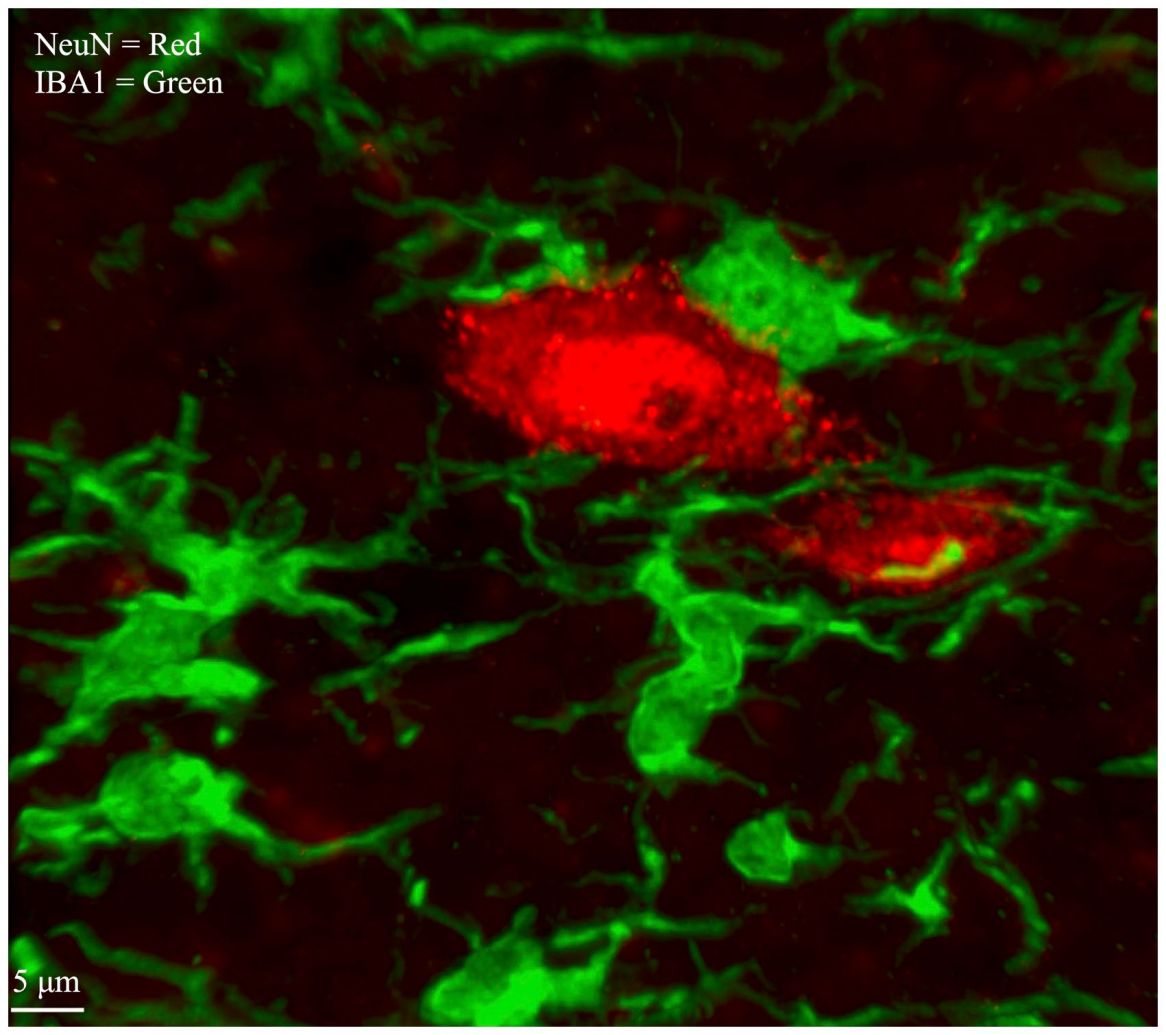
Microglia and Neurons within spinal cord gray matter labeled by multiplex IHC. Neuronal cell bodies, labeled with an antibody that targets NeuN, are visualized with a secondary antibody conjugated to a red fluorophore (Alexa Fluor 568). Microglia, labeled with an antibody that targets ionized calcium binding adaptor molecules (IBA1), are visualized with a secondary antibody conjugated to a green fluorophore (Alexa Fluor 488). Image is taken from laminae IV/V of lumbar enlargement section L3/4, ipsilateral to CCI.

Fluorescent immunohistochemistry (IHC) is a popular method for detection of proteins in fixed tissue (Magaki et al., 2019; Ortiz Hidalgo, 2022). In fluorescent IHC, a fluorophore is conjugated to an antibody directed against a protein of interest on a tissue section. The distribution of the fluorophore within the tissue can then be imaged with an epifluorescence microscope to compare differences in protein expression across treatment groups, or to test for colocalization of multiple proteins within a single tissue sample. Multiple types of proteins can be visualized on the same slice of tissue by using a distinct color of fluorescent reporter for each protein product, a process called multiplexing (Figure 1).

**Figure 2:**
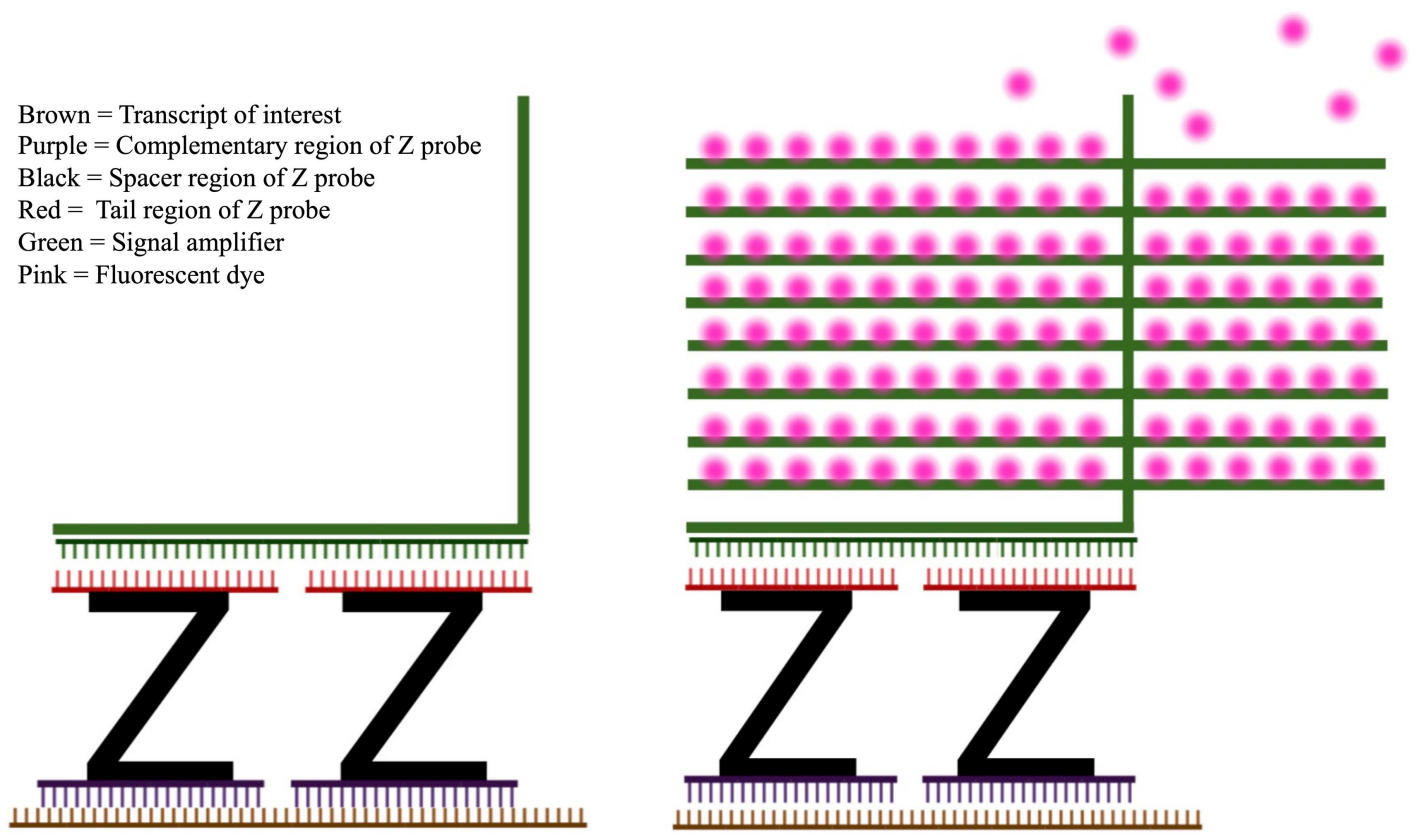
RNAscope Z probe and fluorescence amplifier system. Left: The high specificity and sensitivity of RNA scope is achieved by the design of the Z probes (black) and signal amplifier (green). The lower section of the Z probe (purple) hybridizes with the RNA target of interest (brown). Once two distinct Z probes have hybridized to adjacent sections of the RNA, the amplifier sequence (green) can hybridize across Z probes. Right: After the amplification structure is assembled on top of the Z probe, fluorescent dye (pink) is attached to the amplifier.

An analog of IHC which fluorescently labels RNAs in tissue slices is fluorescent *in situ* hybridization (FISH), where RNAs of interest are hybridized by a complementary strand of nucleic acids called a probe, which is directly or indirectly attached to a fluorescent dye (Young et al., 2020). RNAscope (Advanced Cell Diagnostics, Hayward, CA) is a method of FISH that uses proprietary “Z probes” to minimize off-target fluorescence, as well as a proprietary signal amplification system which attaches to the Z probe to increase the total amount of reporter dye per transcript (Wang et al., 2012). Z probes have a complementary region which hybridizes to the RNA of interest, a spacer region, and a “tail” region which binds to the amplifier structure, which is then labeled with fluorescent dye molecules (Figure 2). RNAscope has superior target specificity because the base of the amplifier structure is designed to bind to a pair of Z probe tails, not to a single Z probe. The hybridization regions of the Z probes are designed in pairs, so that two probes recognize adjacent stretches of the same RNA sequence. Compared to traditional FISH, in which each individual probe is also a fluorescent reporter, a Z probe is only capable of binding to an amplifier and reporting a fluorescent signal when its “partner” Z probe is also bound to the adjacent sequence of the same RNA. Without a pair of adjacent probes hybridizing to the RNA, the fluorescent signal does not occur. Because of this, off-target fluorescence is extremely low (Wang et al., 2012; Atout et al., 2022). This specificity is then combined with a powerful signal amplification step, which attaches a large fluorescent surface area to each Z probe pair by use of the amplification structure. This amplification is powerful enough that a single RNA transcript can be detected with light microscopy (Wang et al., 2012). Both RNAscope and IHC have limitations, which are discussed in detail in Section 4.

In this paper, we present a tissue processing and staining method for thicker (14-μm), fixed spinal cord sections that combines IHC and RNAscope, eliminating the need for RNase-removing reagents. Modifications are made from the default RNAscope protocol to preserve the integrity of spinal cord gray matter and prevent the white matter-rich spinal cord sections from falling off the slide during heat treatment steps. Tissues are fixed at the time of post-mortem collection, sliced in a cryostat at 14-μm, and baked onto the slide after heat treatment and protease steps. Notably, this approach facilitates the assessment of mRNA expression density within each cell type, an important feature that allows for more nuanced analysis of gene expression changes.

After RNAscope and IHC staining, confocal microscopy and image analysis are then used to quantify cell-specific RNA expression, allowing researchers to pinpoint the location of RNA transcripts in relation to specific cell types of interest. To demonstrate the utility of this method, we employed a common model of neuropathic pain, unilateral chronic constriction injury (CCI), which causes large increases in inflammation and activation of immunocompetent cells within the ipsilateral spinal cord dorsal horn relative to contralateral dorsal horn (Hu et al., 2007; Mika et al., 2008). We measured changes in the expression of transcripts for the genes interleukin-1beta (IL-1b) and NLR family pyrin domain containing 3 (NLRP3) (a type of immunological protein which forms protein complexes known as inflammasomes, which trigger inflammation responses), within microglia and neurons of medial laminae IV/V of the lumbar spinal enlargement compared to contralateral control. These inflammatory genes are integral to the innate immune response and are necessary for the maintenance of chronic pain across various animal models, and are also implicated in the pathogenesis of numerous neurodegenerative diseases (Rothwell and Luheshi, 2000; Allan et al., 2005; Ren and Torres, 2009; Ferraccioli et al., 2010). The selection of CCI and laminae IV/V of the lumbar spinal cord for analysis in this study was aimed at demonstrating the efficacy of the methods in detecting changes in gene expression. Despite the high relevance of other laminae, cell types, and genes to pain research, the scope of this methods paper is confined to these two genes and cell types to focus on demonstrating the utility of the method. Using this method, we were able to demonstrate that microglia are largely responsible for increased inflammatory mRNA within the spinal cord, and that total increases in IL-1b and NLRP3 mRNA expression result from both an increase in transcription density within microglia, rather than simply from additional microglia proliferation or recruitment. This efficient, cost-effective method is easy to use with CNS tissue samples, such as those from the spinal cord.

## 2. Methods

### 2.1. Subjects

Male Sprague-Dawley rats (*n* = 6; Inotiv, Indianapolis; 10 weeks of age at arrival) were housed in pairs in an AAALAC approved animal facility on a 12-h light/dark cycle. Rats were allowed to acclimate to colony conditions for 14 days prior to experimental manipulation. All experiments were in accordance with the National Institutes of Health Guide for the Care and Use of Laboratory Animals and were approved by the University of Colorado Boulder Institutional Animal Care and Use Committee. Health checks were conducted daily.

### 2.2. Chronic constriction injury

After acclimation, all six rats received unilateral CCI, as previously detailed (Bennett and Xie, 1988; Grace et al., 2010). All rat received CCI as the analyses to be described will use individual rats as their own controls, rather than comparison to naives or shams. This not only minimizes animal use but is methodologically advantageous as it: (a) guards against noise in sampling across groups, as a single tissue slice serves as both the ipsilateral section and its own contralateral control; and (b) using the contralateral side as the control requires half the costs in reagents, animal lives, and microscopy time.

Briefly, CCI was aseptically performed under inhalant anesthesia (3% aerosolized isoflurane). After gentle isolation of the sciatic nerve at mid-thigh level, it was loosely ligated with four sterile chromic gut sutures (4-0 chromic gut; Patterson Veterinary, Devens, MA). After CCI, rats were monitored postoperatively until fully ambulatory before returning to their home cage. As is standard for the CCI model, no anti-inflammatories or analgesics were given after surgery as this can alter the immune and glial underlying processes that generate the neuropathic state. As standardly observed with CCI, with from the first description of this model, the paw ipsilateral to CCI (but not control) is held in a guarded position, supportive of a successful surgery (Bennett and Xie, 1988).

### 2.3. Tissue collection and preparation

Tissues were collected 7 days after CCI, as CCI-induced mRNA changes in spinal cord dorsal horn are already robust at this point (Latrémolière et al., 2008; Mika et al., 2008; Jurga et al., 2015). A detailed protocol for tissue collection and processing is included in Supplementary Material S1. At the time of time of tissue collection, rats were given a lethal dose of sodium pentobarbital followed by transcardial perfusion with ice-cold 0.9% saline in deionized water (DI) until the liver appeared cleared of blood, and ice-cold 4% paraformaldehyde in 0.1 M phosphate buffered water (PB) (pH 7.4) for 4 min. Lumbar spinal cords were then isolated and post-fixed by immersion in 4% paraformaldehyde/PB (pH 7.4) for 4 h at 4°C. Section 4 discusses the length of post-fixation time as a variable that may need to be adjusted depending on the research context. Post-fixation time can impact the amount of protease used and, therefore, the extent of destruction of protein epitopes. For our 14 μm thick spinal cord sections, a post-fixation time of 4 h was sufficient to maintain tissue integrity and achieve clear labeling of both mRNA and proteins of interest after protease treatment. However, it is crucial that all samples undergo post-fixation for the same amount of time, regardless of the total duration. Failure to do so can result in high variability in RNA labeling.

Spinal cords were then cryoprotected across sequential days (24 h per solution) at 4°C with 15, 20, and 30% sucrose in 0.1 M PB (pH 7.4). Lumbar spinal cords were then freeze-mounted in Tissue-Tek optimal cutting temperature compound (OCT). Although the manufacturer’s default protocol recommends fresh, unfixed sections of 8 μm in thickness to avoid loss of tissue during the heating and protease steps, we were able to successfully label fixed sections up to 14 μm in thickness with no tissue loss by re-adhering the spinal cord sections to the slides after heated target retrieval, as discussed in Section 2.4. Frozen sections of 14 μm thickness were cut in a cryostat at −18°C and collected onto SuperFrost+ slides (Thermo Fisher Scientific, Hampton NH).

The slides were allowed to dry completely at room temperature (RT) for 1 h prior to storage at −80°C. Noteworthy regarding this procedure is that sections thicker than 14 μm are not recommended as we found that 20 μm spinal cord sections do not adhere to the slides, even with the additional baking step described above.

### 2.4. RNAscope + immunohistochemistry

A detailed protocol for RNAscope + IHC staining is included in Supplementary Material S1. The slide mounted tissue sections were prepared for RNAscope probe hybridization through a series of steps including dehydration, peroxide blocking, and target retrieval blocking. Briefly, slides were first selected at random so that an unbiased tissue section from the lumbar enlargement of each animal (L3-6) was represented. The tissue was then dehydrated via 5-min incubations across a gradient of 50, 75, 100, and 100% molecular grade ethanol, and then serially incubated in RNAscope H_2_O_2_ from ACDbio for 15 min at room temperature followed by RNAscope Target Retrieval reagent (ACDbio) for 5 min at 98°C. Because tissue sections such as spinal cord that are thick and rich in white matter tend to fall off slides when following the manufacturer’s default protocol, it is critical that tissue sections are baked dry at 67°C for 30 min upon completion of RNAscope Target Retrieval. Slides were then incubated in Protease III solution (ACDbio) at 40°C for 15 min. While other proteases are included in the kit, Protease I and II were found to be destructive to epitopes of interest, impairing later antibody labeling. The mRNA in the tissue was hybridized by incubating in RNAscope buffered Z probes for either NLRP3 (ACDbio ref. 510041) or IL-1 (ACDbio ref. 314,011-C2) for 2 h at 40°C and stored overnight in 5x saline sodium citrate.

The next day, slides were incubated in RNAscope amplifier components and tagged with a fluorescent dye, as specified in the manufacturer’s instructions. Specifically, the slides were sequentially incubated in AMP1 (ACDbio) for 30 min at 40°C, AMP2 (ACDbio) for 30 min at 40°C, and then AMP3 (ACDbio) for 15 min at 40°C. The attached RNAscope amplifier structure was opened with a channel-specific horseradish peroxidase (ACDbio) to allow fluorescent labeling. The tissue was then incubated with the far-red dye Opal650 (Akoya Bio, Delaware) for 30 min at 40°C and treated with channel specific horseradish peroxidase blocker (ACDbio) to close the amplifier structure.

Immediately after fluorescent RNA labeling, tissue sections were incubated overnight at 4°C in primary antibodies in solution with Co-Detection Diluent (ACDbio). The primary antibodies used were: 1:250 mouse anti-NeuN (EMD Millipore Ref MAB377) and 1:1000 guinea pig anti-IBA1 (Synaptic Systems Ref 234004). After overnight incubation, the tissue was gently washed in DI water and then incubated with secondary antibody solutions for 2 h. The secondary antibodies used were: 1:200 goat anti-guinea pig 488 (Invitrogen Ref A11073) and 1:200 goat anti-mouse 568 (Invitrogen Ref A11004), diluted in Co-Detection Diluent. Finally, the slides were washed in DI water and coverslipped with ProLong Glass mounting medium (Thermo Fisher Scientific, Hampton NH).

### 2.5. Microscopy

**Figure 3:**
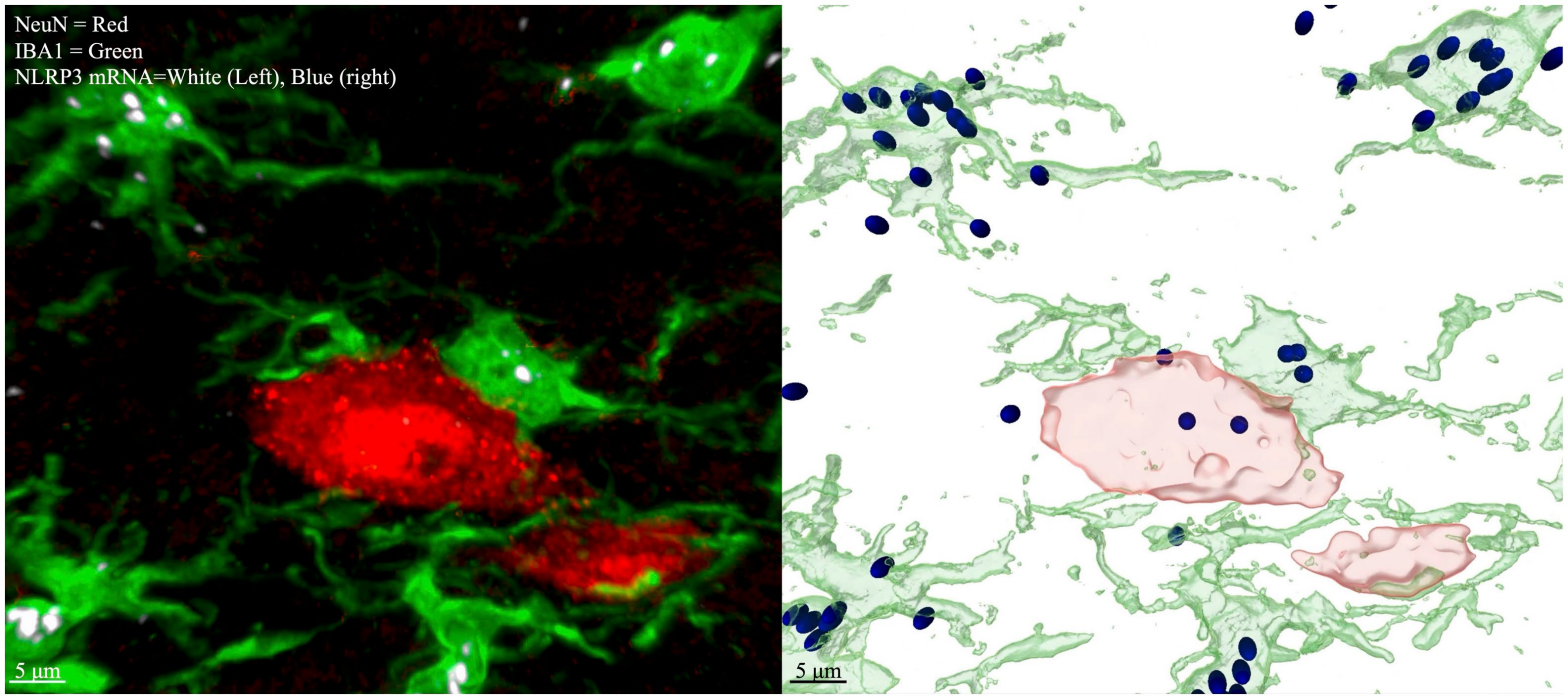
Multichannel IHC with labeled NLRP3 mRNA (left), Imaris modeling (right). Red = NeuN (neurons), Green = IBA1 (microglia), White (left) and Blue (right) = NRLP3 mRNA. Image is taken from laminae IV/V lumbar enlargement ipsilateral to CCI. Spots are rendered by Imaris if the fluorescence signal meet an intensity threshold over background, so dim spots that are at a similar level of fluorescence intensity to nondescript tissue background will not be counted as spots or seen on the rendered image (right). The same intensity threshold for puncta applied to all images in the dataset.

**Figure 4:**
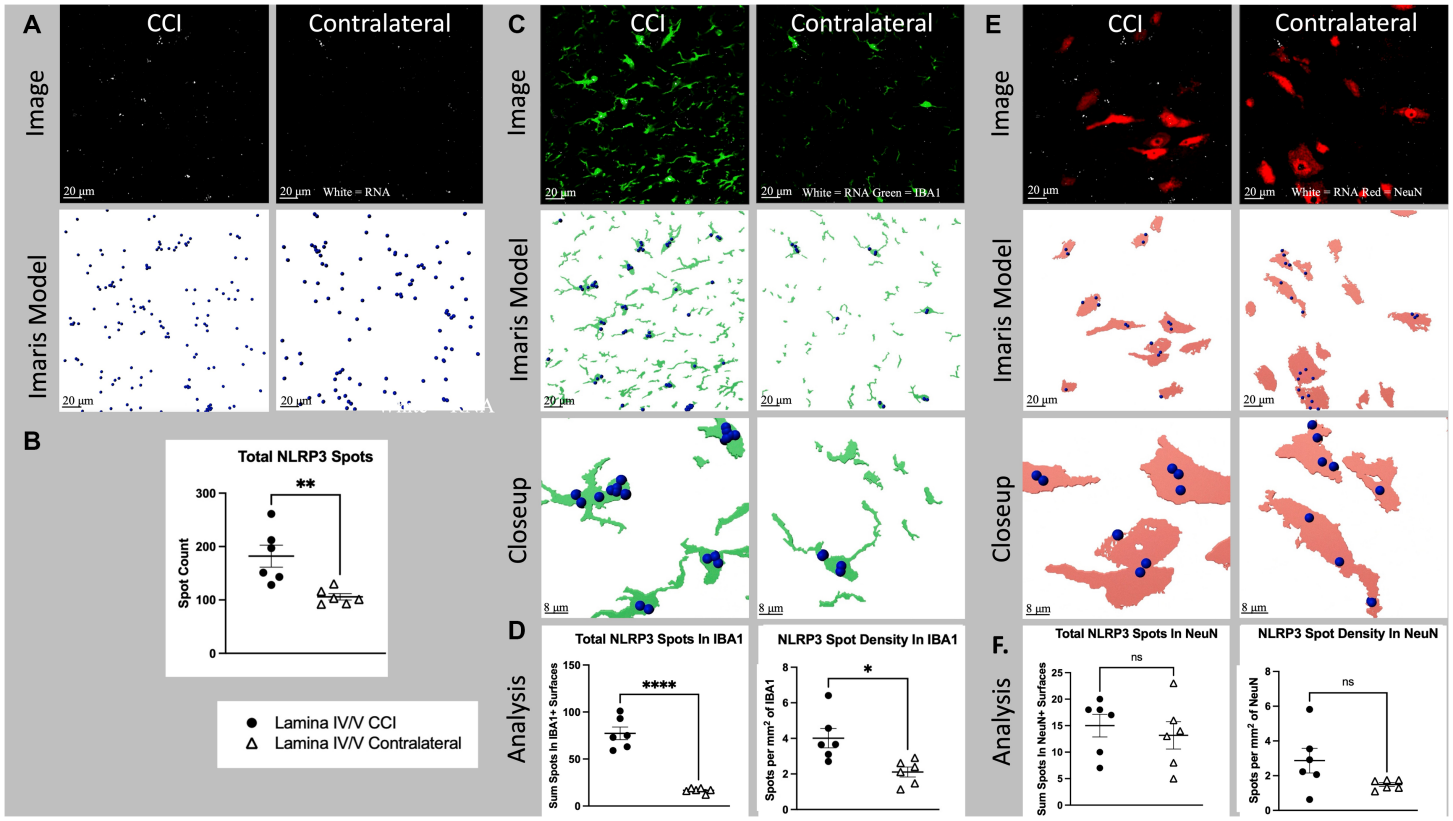
Cell specific NLRP3 RNA expression in lamina IV/V lumbar (L3-6) dorsal horn ipsilateral to CCI vs. contralateral control. **(A)** Total NLRP3 spots (white) CCI vs. contralateral. Top images show a full, single field of view confocal image taken from the medial aspect of lamina IV/V (see Supplementary Figure S1 for region sampled). Bottom images show Imaris spot modeling (size of spots increased 4× for visualization). Total spots represent all labeled fluorescent puncta in a single field of view which meet brightness (intensity) threshold for inclusion in the RNA count. **(B)** Total NLRP3 spots for all animals CCI vs. Contralateral (*p* = 0.0053). **(C)** NLRP3 (white) colocalized with IBA1 (green), CCI vs. contralateral. Top images are the same as Figure 5A, but with green IBA1 channel also shown. Middle images show Imaris models of IBA1+ surfaces colocalized NLRP3 spots. Bottom images show a closeup of Imaris models. **(D)** Light shows total spots within IBA1+ surfaces in a single field of view (*p* < 0001). Right shows density of NLRP3 spots within IBA1+ surfaces (*p* = 0.0110). **(E)** NLRP3 (white) and NeuN (red), CCI vs. contralateral. Top images are the same as Figure 5A, but with red NLRP3 channel also shown. Middle images show Imaris models of NeuN+ surfaces and colocalized NLRP3 spots. Bottom images show a closeup of Imaris models. **(F)** Left shows total spots within NeuN+ surfaces in a single field of view (*p* = 0.5952). Right shows density of NLRP3 spots within NeuN+ surfaces (*p* = 0.0847). Graphs show individual datapoints (*N* = 6) and SEM. Each datapoint on subfigures **(B,D,F)** represents a single image from laminae IV/V of a single tissue section taken randomly from lumbar enlargement (L4-6) from an individual rat.

**Figure 5:**
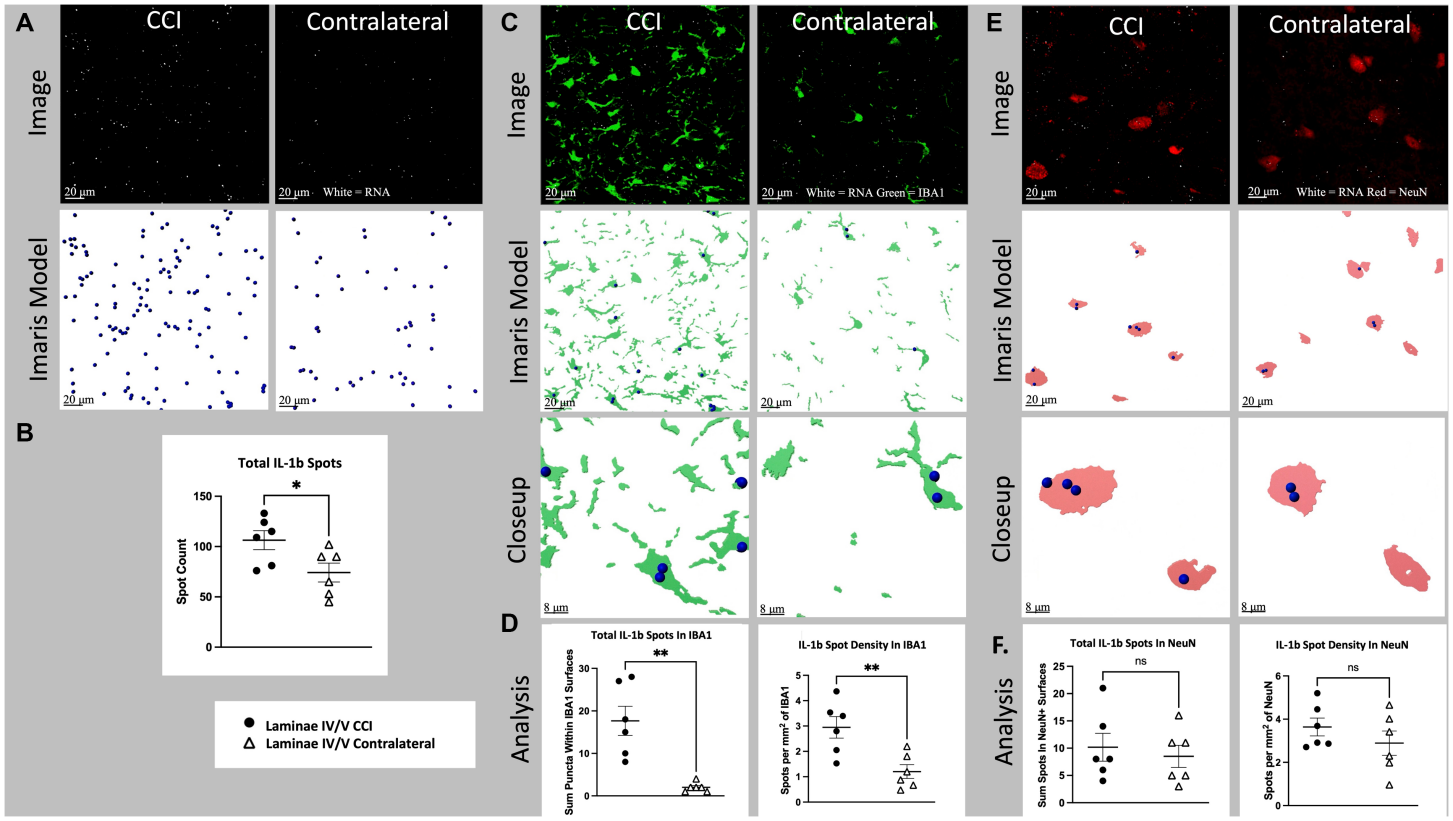
Cell specific IL-1b RNA expression in lamina IV/V lumbar (L3-6) dorsal horn ipsilateral to CCI vs. contralateral control. **(A)** Total IL-1b spots (white), CCI vs. contralateral. Top images show a full, single field of view confocal image taken from the medial aspect of lamina IV/V (see Supplementary Figure S1 for region sampled). Bottom images show Imaris spot modeling (size of spots increased 4× for visualization). Total spots represent all labeled fluorescent puncta in a single field of view which meet brightness (intensity) threshold for inclusion in the RNA count. **(B)** Total IL-1b spots for all animals CCI vs. contralateral (*p* = 0.0364). **(C)** IL-1b (white) and IBA1(green) CCI vs. contralateral. Top images are the same as Figure 6A, but with green IBA1 channel also shown. Middle images show Imaris models of IBA1+ surfaces and colocalized Il-1b spots. Bottom images show a closeup of Imaris models. **(D)** Left shows total spots within IBA1+ surfaces in a single field of view (*p* = 0.0011). Right shows density of IL-1b spots within IBA1+ surfaces (*p* = 0.0065). **(E)** *il*-1b (white) and NeuN (red), CCI vs. contralateral. Top images are the same as **(A)**. but with red NLRP3 channel also shown. Middle images show Imaris models of NeuN+ surfaces and colocalized IL-1b spots. Bottom images show a closeup of Imaris models. **(F)** Left shows total spots within NeuN+ surfaces in a single field of view (*p* = 0.6211). Right shows density of IL-1b spots within NeuN+ Surfaces (*p* = 0.3094). Graphs show individual datapoints (*N* = 6) and SEM. Each datapoint on subfigures **(B,D,F)** represents a single image from laminae IV/V of a single tissue section taken randomly from lumbar enlargement (L3-6) from an individual rat.

Images for Figures 1, 3–5 were acquired using a Nikon A1R model laser scanning confocal microscope with Nikon elements software (v5.30.05). A Z stack image was used to show the stain in detail with complete cell morphology (Figures 1, 3). The images acquired for analysis (presented in Figures 4, 5) were 2D single confocal Z slices from the central optical plane of the stained tissue. Images were obtained from a single tissue section from each animal (*N* = 6), randomly chosen from sections collected within lumbar levels 3–6 (L3–L6) as CCI-induced changes are anticipated throughout these spinal levels. For each tissue section, a Nikon 60×/1.4NA oil objective was used to image a single field of view of ~220 × 220 μm of the medial gray matter of laminae IV/V in the dorsal horn, both ipsilateral and contralateral to the CCI site. The region chosen for analysis (depicted in Supplementary Figure S1) represents part of the somatotopic map related to the digits and plantar surface of the hind paw, regions which become chronically sensitive to touch in the CCI model (Woolf and Fitzgerald, 1986). While other areas of the spinal cord are relevant to chronic pain and the CCI model, a comprehensive exploration of pain-related neuroinflammatory phenomena falls beyond the purview of this paper. Image parameters were acquired with 1,028 × 1,028 XY steps using a pixel size of 0.21 μm/pixel. Lasers used were 488 nm, 561 nm, and 638 nm, paired with emission filters of 525/50, 600/50, and 685/70, respectively. The three-dimensional Z-stack image used in Figures 1, 3 was acquired with 27× Z steps 0.2 μm in size.

**Figure 6:**
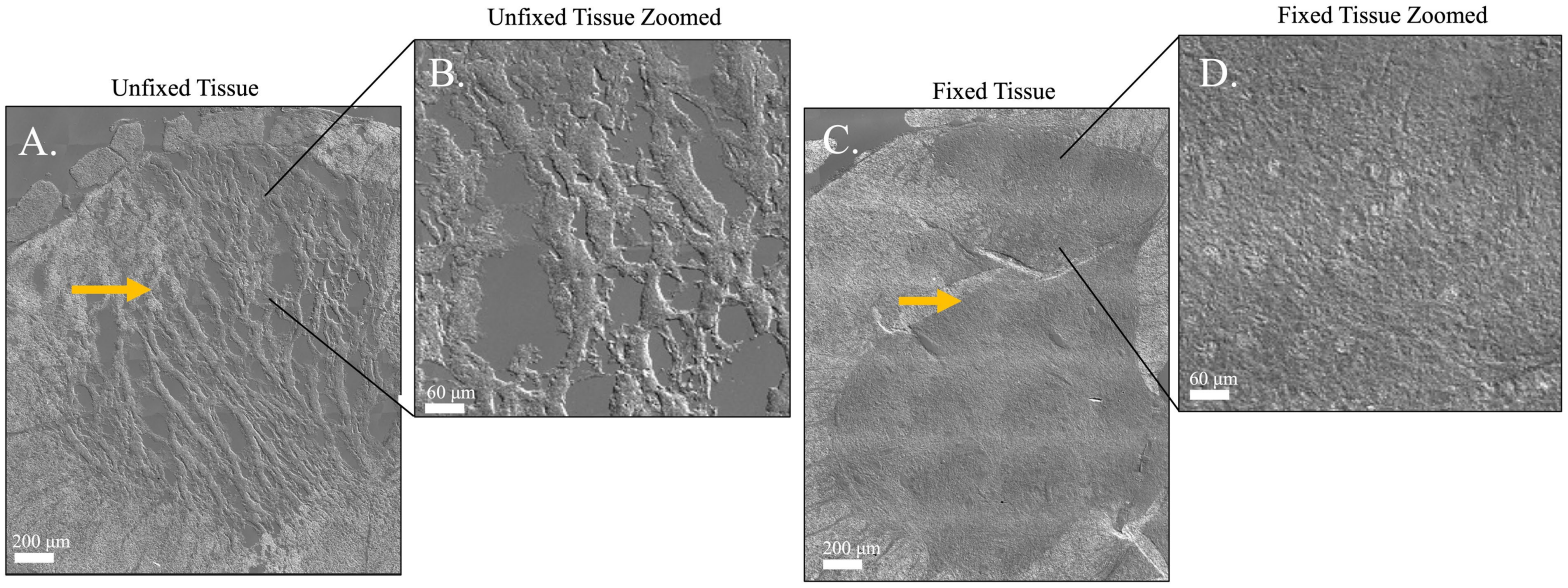
Differential interference contrast images comparing tissue integrity of two spinal cord sections which have been processed for RNAscope + IHC either by the default recommendations included in the RNAscope multiplex kit **(A,B)** or the methods described in this paper **(C,D)**. **(A)** An 8 μm thick unfixed spinal cord section from an early attempt at combining RNAscope + IHC in spinal cord. **(B)** Zoom of inset shows damage to gray matter. Notice the torn gaps in the tissue. **(C)** A 14 μm thick fixed spinal cord section with intact gray matter, processed as described in this methods paper. **(D)** Zoom of inset shows improved tissue integrity after fixation and thicker sectioning. The unfixed gray matter is prone to tearing after fresh flash freezing and cryostat sectioning. Due to extensive tearing in the unfixed tissue, the spatial boundary where gray matter turns to white matter is ragged and undefined but remains crisp and clear for image analysis in the thicker, fixed tissue (Yellow arrows). Scale Bar = 200 μm **(A,C)** or 60 μm **(B,D)**. These images are of sections from a pilot experiment comparing fixed vs. non-fixed tissues and not included in the analyses reported in this manuscript.

Figure 6 was acquired on a Nikon eclipse widefield with Nikon elements software (v5.30.05) and a Hamamatsu ORCA Fire CMOS camera. A 20× mosaic using differential interference contrast (DIC) imaging was acquired to highlight torn edges of the tissue.

### 2.6. Image analysis

Standard image analyses were undertaken to colocalize the RNAscope puncta and the labeled cell bodies using Imaris software (Oxford Instruments v10.1). Briefly, RNAscope puncta above a brightness threshold were marked. The spatial position (X and Y values) for each marked puncta was entered into a database. The image channels for each IHC stain were analyzed independently. The spatial values for IBA1+ areas of the image were used to model microglia cells, the NeuN+ areas of the image were used to model neuronal somas. The spatial values of these models served as the spatial position of the labeled cells modeled by the IHC stains. By comparing the locations of the RNA puncta and the boundaries of each cell, RNA counts were subdivided by cell type, and normalized by total cell area sampled to assess spatial density of labeled RNA puncta. Imaris software modules were used for these functions as well as basic image processing algorithms for enhanced edge detection, reduced background noise, etc. For RNAscope + IHC analysis on Imaris, RNA scope puncta were marked with the Imaris “Spots” module, and the IHC stains which delineate cell boundaries (for example, NeuN and IBA1) were processed with the Imaris “Surface” module, which generates a model of the boundaries of each cell. These boundaries were used to both define a region of interest (ROI) and normalize to the total area/volume of the ROI (Figure 3). Similar analysis methods can be achieved with ImageJ and Matlab, though the difficulties of building accurate but flexible algorithms and managing multiple databases for quantifying the spatial relationships of objects in Matlab make it beyond the scope of this paper (Gautier and Ginsberg, 2021).

### 2.7. Statistical analysis

Unpaired *t*-tests were used to determine RNA expression, measured as the total number of labeled RNA puncta within a single field of view, compared between laminae IV/V of ipsilateral and contralateral dorsal horn. Total RNA expression in neurons and microglia was measured as total number of labeled RNA puncta which colocalize with NeuN or IBA1, respectively. Density of neuronal NLRP3 expression was measured as the total labeled RNA puncta which overlap with NeuN divided by the total NeuN+ area. The parallel calculation was done for microglial NLRP3 relative to IBA1. Differences in labeling in laminae IV/V of the dorsal horn ipsilateral vs. contralateral to CCI were compared with unpaired *t* tests on each comparison listed above. Values were graphed as individual data points and standard error of mean (SEM). All analyses were performed using Prism 9 (GraphPad, CA, United States). *p* < 0.05 was considered statistically significant.

## 3. Results

### 3.1. Nerve injury significantly increases both the overall and microglia-specific NLRP3 mRNA expression in spinal cord dorsal horn laminae IV/V, with no impact on neuron-specific expression

Nerve injury (CCI) significantly increased laminae IV/V NLRP3 mRNA expression in ipsilateral compared to contralateral dorsal horn (*p* = 0.0053; Figures 4A,B). Within IBA1+ regions of laminae IV/V, NLRP3 mRNA was increased ipsilaterally compared to contralateral (*p* < 0.0001), as was the density of NLRP3 mRNA contained within modeled microglia (*p* < 0.0110; Figures 4C,D). The elevated mRNA density in modeled microglia indicates that the heightened NLRP3 mRNA expression is due to increased per-cell transcription, not merely from additional microglia proliferation or recruitment. CCI did not significantly increase either NLRP3 mRNA within NeuN+ regions (*p* = 0.5952), or the density of NLRP3 within NeuN+ regions of laminae IV/V compared to the contralateral site in dorsal horn (*p* = 0.0847; Figures 4E,F).

### 3.2. Nerve injury significantly increases both the overall and microglia-specific IL-1b mRNA expression in spinal cord dorsal horn laminae IV/V, with no impact on neuron-specific expression

Nerve injury (CCI) significantly increased laminae IV/V IL-1b mRNA expression in ipsilateral compared to contralateral dorsal horn (*p* = 0.0364; Figures 5A,B). Within IBA1+ regions of laminae IV/V, IL-1 mRNA was increased ipsilaterally compared to contralateral (*p* = 0.0011), as was the density of IL-1 (*p* = 0.0065; Figures 5C,D). The increased mRNA density suggests that some of the difference in expression of IL-1 mRNA is due to increased transcription per cell, rather than being accounted for by proliferation and/or recruitment of additional microglia. CCI did not significantly increase either IL-1 mRNA within NeuN+ regions (*p* = 0.6211), or the density of IL-1 within NeuN+ regions of laminae IV/V compared to the contralateral site in dorsal horn (*p* = 0.3094; Figures 5E,F).

## 4. Discussion

Both RNAscope and IHC have limitations on their own. Accurate, clear IHC labeling depends on the sensitivity and specificity of the antibody used to label a protein of interest. For some immunological proteins, high-quality antibodies suitable for use in tissue IHC can be rare, as the antibodies may fail to target the antigen of interest exclusively or even primarily, muddying signal of interest with signal from off-target proteins (Rhodes and Trimmer, 2006; Fritschy, 2008). A notable example is NLRP3, which one study found that eight of eight commercially available antibodies failed tests of sensitivity, specificity, or both (Kosmidou et al., 2001). Further, specific antibody labeling reported in cell culture is not guaranteed to retain specificity in fixed tissue slices, which include many more off-target epitopes from the variety of cell types, fixation-induced changes in epitopes, and tissue matrix that can compete with the epitope of interest (Bogen et al., 2009; Scalia et al., 2017). Projects such as the nonprofit NeuroMab (University of California, Davis) exist solely to produce and validate antibodies in neural tissue, as nonspecific antibodies have contributed to false positives and replication crises (Fritschy, 2008; Gautron, 2019; Dalmau, 2020). Non-specific staining is especially problematic when fluorescent target labels appear as small, physically nondescript dots, and thus not morphologically distinguishable from nonspecific noise. On the other hand, the two principal weaknesses of RNAscope alone when performed as recommended by the manufacturer (ACDbio, 2023) are the inability to visualize cell morphology and instruction to use fresh, unfixed tissue. Because RNAs are neither primary components of the cytoskeleton nor abundant along the outer cell membrane, RNAscope cannot be used to visualize cell morphology with the fidelity of IHC. An additional downside for spinal cord is that the default RNAscope protocol calls for tissue to be collected, frozen, and thinly sliced (8-μm) onto slides without fixation. While fresh collection is not a problem for some tissues, spinal cord gray matter is easy to tear when slicing on a cryostat, especially when unfixed and thinly sectioned (compare Figures 6A,B to Figures 6C,D). The use of unfixed tissue also requires that the tissue be collected and processed using strictly RNase-free equipment, necessitating the use of RNase-eliminating reagents prior to tissue processing.

We demonstrated RNAscope and IHC combined in single sections of fixed spinal cord tissue. The process of combining these two techniques is optimized for thicker (up to 14-μm) tissue sections, allowing assessment of RNA expression within full modeled cells in delicate tissues, including those containing considerable white matter (i.e., fats) which increases the likelihood of tissue slices falling off the slides during processing. *In situ* hybridization in general, and RNAscope specifically, is standardly done in tissue that has been quickly extracted and slide mounted without fixation or perfusion (Lammers et al., 2001; ACDbio, 2023). However, fixation does not destroy RNA nor does it render RNA permanently inaccessible. RNAs can still be detected in formalin-fixed tissues for weeks after extraction at similar levels to fresh tissues with the aid of proteases and heat to retrieve RNA sequences (Lehmann and Kreipe, 2001; van Maldegem et al., 2008; McKinney et al., 2009). Indeed, several papers have used RNAscope in formalin fixed ovarian tissue as well as combined RNAscope + IHC in fixed brain and peripheral nerve (Bingham et al., 2017; Villapol et al., 2017; Li et al., 2021). The experiments included here have shown that tissue which has been fixed by transcardial perfusion with a 4-h post-fixation in 4% paraformaldehyde before blocking can be used for highly sensitive and robust RNA plus IHC stains that capture differences in gene expression after nerve injury. As shown in many prior experiments using PCR to analyze RNA from saline perfused animals, much RNA remains in tissue without the use of DEPC treated water (Frank et al., 2006; Green-Fulgham et al., 2022; Zhang et al., 2022). Cold formalin preserves nucleic acid integrity, so the solution of 4% paraformaldehyde was chilled and kept on ice throughout the perfusions (Bussolati et al., 2011).

One advantage of fixing tissue at the time of collection, prior to sectioning, is the ability to bypass the time-consuming and costly preparation of tools and workspace to maintain an RNase-free environment. While freshly collected, unfixed tissue is vulnerable to environmental RNases and contains active endogenous RNases, formaldehyde fixation offers substantial protection for RNAs, inactivates endogenous RNases, and reduces the need for strict RNase-free sample preparation. The electrophilic nature of formaldehyde and paraformaldehyde allows it to form cross-links between proteins and nucleic acids by covalently bonding to charged residues in proteins and nucleases, including amino groups of lysine residues, imidazole groups of histidine residues, and thiol groups of cysteine residues (Fox et al., 1985; Dapson, 2007; Kim et al., 2017). This leads to the formation of methylene bridges between amino groups on nucleic acids and cross-linking between RNAs and proteins, ultimately preserving RNA (Srinivasan et al., 2002). As mentioned earlier, RNA in tissue that has been exposed to room temperature formaldehyde for weeks is preserved (Lehmann and Kreipe, 2001; van Maldegem et al., 2008; McKinney et al., 2009). Moreover, the enzymatic activities of RNases and other enzymes acting on RNA, such as reverse transcriptases, have been shown to be significantly reduced or even completely eliminated in fixed tissue under laboratory conditions (Jonsson and Lagerstedt, 1957, 1959; von Ahlfen et al., 2007). Therefore, formaldehyde fixation not only provide structural support for delicate tissue but also allows for the slicing and slide mounting process to be conducted without strict RNase-free conditions.

The ability to use RNAscope + IHC on thicker (14-μm), fixed tissues is critical when working with delicate tissue sections that may tear when exposed to shear forces of cryostat blades, such as spinal cord (Figure 6). By fixing delicate tissues prior to slicing at 14 μm thickness, tissue integrity is better preserved. In our experience, 14-μm-thick spinal cord tissue sections tended to detach from the slide, but incorporating a heating and drying step prior to protease treatment effectively prevents tissue loss throughout the procedure. The duration of fixation and strength of subsequent protease treatments should be considered variables to be systematically varied to define optimal parameters for the analytes under test, rather than absolute restrictions for RNAscope assays. However, the advantages of fixation must be weighed against the costs of subsequent protease and heat treatments, as well as the potential loss of absolute signal values on either mRNA or protein. There is a significant gap in literature when it comes to quantifying alterations in probe target specificity post-fixation, both for RNAscope and conventional *in-situ* hybridization methods. The literature lacks experiments that quantify changes in target probe target specificity after fixation, both for RNAscope and traditional *in situ* hybridization methods with fluorescently labeled probes. While probe specificity cannot be proven without a knockout model of the genes of interest, off-target background staining can be estimated is to use an off-target negative control probe which labels an bacterial RNA not found in your model species. Although this control will not prove the probe of interest’s specificity, it can provide an estimate of background staining. In the original RNAscope paper by Wang and colleagues, the RNAscope system was tested on a variety of fixed tissues, including up to 24 h of fixation, in which “completely negative results were often found” (Wang et al., 2012). The specificity is likely due to the strength of the RNAscope Z probe system, where two physically independent Z probes must hybridize to adjacent sections of the same transcript for the amplifier and then fluorescent reporter to stably bind. The use of a light protease (Protease 3, ACD bio) prior to RNA probe hybridization allows the RNAs to be accessed while preserving enough protein antigen for IBA1 and NeuN IHC labeling of microglia and neurons, respectively.

By using RNAscope, one can only determine the co-localization of genes of interest with other RNAs inside a cell. However, the combination of RNAscope with IHC allows for more accurate localization of mRNAs than *in situ* hybridization alone. The IHC technique provides sharp spatial boundaries for each cell type, enabling the labeling of both cell boundaries and RNAs to measure the spatial density of RNAs within cells. *In situ* hybridization alone cannot accomplish this. NRLP3 and IBA1 puncta were mostly localized within the cell somas, but were occasionally found in the processes of the microglia. This kind of nuanced observation about the location of RNAscope labeled puncta within the cell is not possible without a model of the cell morphology provided by IHC.

Although the tissue preparation steps for optimal IHC and RNAscope diverge, the powerful signal amplification of the RNAscope method provides a clear, strong signal even when using fixed tissue sections that are thicker than standard specifications. The use of protease treatment on the tissue sections does reduce the signal of IHC, so the method will work best when the proteins of interest are highly expressed and/or resistant to protease degradation. Interestingly, in our antibody tests, we found that IBA1 remains abundant and bright after protease processing. NeuN seemed resistant to the protease processing, though the brightness of the fluorescent signal was noticeably weaker (qualitative impression as roughly twice as bright as background fluorescence) compared to the same tissue processed for IHC alone (qualitative impression as roughly four times as bright as background fluorescence). These non-quantitative comments are included simply to relay that NeuN was still a sufficiently strong signal for analysis, but was weakened by the protease processing.

A point worth considering here is that protein degradation within cells has the potential to affect the results of RNA localization. That is, logically, proteins for cells of interest (for example, cell-type specific markers) may be degraded enough that only a subset of those cells are visualized by IHC. If this were true for a given cell it would be a false negative when analyzing which cell types express the RNA of interest. Therefore, it cannot be assumed that because an RNA has failed to be located inside of a cell specific IHC marker that the RNA must be in another cell type, without checking to see if protein degradation has impacted the ability to fully model the cells of interest in the tissue region. This consideration is in addition to general limitations of IHC markers for cell specific staining. For example, NeuN only labels the center of the soma, failing to label the lateral aspects of the soma, dendrites, and axons of a neuron, all of which may contain RNAs. Cell specific RNA quantification using IHC + RNAscope should be considered as a sample of the specific cell type, rather than the entire population of that cell type.

The ability to combine IHC and RNAscope labeling in fixed, thicker sliced tissue is valuable for any work with delicate tissues prone to tearing, or where RNAs must be quantified throughout a cell with branching morphology. The use of RNA probes where quality commercial antibodies are not available offers the ability to measure gene expression changes *in situ*. By co-localizing with fluorescent IHC using cell-specific markers or other cellular proteins of interest, changes in RNA expression in selected cell types can be quantified. Results shown above demonstrate the potential of this technique, such as detection of increased NLRP3 and IL-1 in ipsilateral dorsal horn after CCI and the identification of microglia, not neurons, as a major source of these increases. Changes in mRNAs for NLRP3 and IL-1 in spinal cord have been reported in prior publications but have relied on PCR from grossly dissected samples or *in-situ* hybridization alone without full cell morphology modeling (Prins et al., 2013; Grace et al., 2016; Chen et al., 2018). Although this methods paper does not incorporate a comprehensive analysis of relevant dorsal horn laminae, initial observations suggest a similar effect within laminae I/II, a nociceptive specific portion of the gray matter which also has robust gliosis after CCI. While a detailed exploration of CCI-induced inflammation throughout the dorsal horn is beyond the scope of this methods paper, initial inspection suggests that this pattern of inflammation may be generalizable across other areas in the ipsilateral dorsal horn. Consequently, the methods detailed in the paper could provide valuable insights in future pain-related studies.

## Data availability statement

The raw data supporting the conclusions of this article will be made available by the authors, without undue reservation.

## Ethics statement

The animal study was approved by Institutional Animal Care and Use Committee (IACUC), University of Colorado Boulder. The study was conducted in accordance with the local legislation and institutional requirements.

## Author contributions

JB performed experiments, analyzed data, and wrote the manuscript. CM, DR, and EP optimized RNA + IHC labeling. SG-F performed experiments and analyzed data. JD contributed to imaging and image analysis. IC and MF performed pilot experiments and image analysis. IS and BW assisted with tissue sectioning and image analysis. LW, MB, and DR edited the manuscript. All authors contributed to the article and approved the submitted version.

## Funding

This work was supported by National Institutes of Health grants R01 AT009564 (LW), R01 DA044934 (LW), and R21 MH116353 (MB).

## Conflict of interest

The authors declare that the research was conducted in the absence of any commercial or financial relationships that could be construed as a potential conflict of interest.

## Publisher’s note

All claims expressed in this article are solely those of the authors and do not necessarily represent those of their affiliated organizations, or those of the publisher, the editors and the reviewers. Any product that may be evaluated in this article, or claim that may be made by its manufacturer, is not guaranteed or endorsed by the publisher.
