# Supplementary material for: Combining RNAscope and immunohistochemistry to visualize inflammatory gene products in neurons and microglia
Source: Front Mol Neurosci. 2023 Aug 17;16:1225847. doi: 10.3389/fnmol.2023.1225847 (PMC10470653; doi:10.3389/fnmol.2023.1225847)
Supplement: Supplementary file 1 [file Data_Sheet_1.docx]

Supplementary Material S1: Protocol for Tissue Processing and Staining

A. Reagents Required

1. Ice-cold 0.9% saline solution
2. Ice-cold 4% paraformaldehyde in 0.1M phosphate buffer (PB) solution
3. 15%, 20%, and 30% sucrose in 0.1M PB solutions
4. Optimal cutting temperature (OCT) compound (Sakura Finetek, Torrance, CA Catalogue# 4583)
5. Cryomolds (Sakura Finetek Catalogue# 4557)
6. RNAscope target retrieval reagent (ACDbio, Newark, CA Catalogue# 323100)*
7. Saline sodium citrate buffer (SSC) (Invitrogen, Vilnius, Lithuania Catalogue #AM9770)
8. Ethanol solutions: 50%, 75%, and 100%
9. RNAscope hydrogen peroxide solution (ACDbio Catalogue# 323100)*
10. RNAscope Protease III (ACDbio Catalogue# 323100)*
11. RNAscope probes (ACDbio)
    1. NLRP3 (ACDbio ref 510041)
    2. IL1b - (ACDbio ref 314011-C2)
12. RNAscope wash buffer (ACDbio Catalogue# 310091)
13. RNAscope AMP 1, AMP 2, and AMP 3 reagents (ACDbio Catalogue# 323100)*
14. RNAscope channel specific Horseradish Peroxidase (HRP) (ACDbio Catalogue# 323100)*
15. Fluorescent reagents (Akoya Biosciences (Malborough, MA)
    1. Opal 650nm Reagent Kit (Akoya Biosciences Catalogue# 1496001KT)
16. TSA Buffer (ACDbio Catalogue# 322809)
17. RNAscope channel specific HRP-blockers (ACDbio Catalogue# 323100)*
18. Primary antibodies
    1. Mouse anti-NeuN (EMD Milipore Catalogue# MAB377
    2. Guinea pig anti-IBA1 (Synaptic Systems Catalogue# 234004)
19. Co-detection diluent (CDD) (ACDbio Catalogue# 323160)
20. Secondary antibodies
    1. goat anti-guinea pig 488 (Invitrogen Catalogue# A11073)
    2. goat anti-mouse 568 (Invitrogen Catalogue# A11004)
21. DAPI solution (ACDbio Catalogue# 323100)*
22. Prolong mounting medium
    1. Prolong Glass mounting medium for oil immersion objectives (Invitrogen Catalogue# P36984)
    2. Prolong Diamond mounting medium for air objectives (Invitrogen Catalogue# P36965
23. Distilled water
24. 0.1M PBS (Phosphate-buffered saline)

*Reagent is included in the ACDbio RNAscope multiplex fluorescent reagent kit v2, Catalogue# 323100

B. Protocol

1. Tissue Collection and Blocking

1.1. Cardiac Perfusion:

- Perform cardiac perfusion on the animals, first with ice-cold 0.9% saline solution until the liver is clear to flush the blood from the tissues, followed by a 4-minute perfusion with chilled 4% paraformaldehyde prepared in 0.2M phosphate buffer (PB) solution.

1.2. Postfixation:

- Collect tissue of interest and postfix them by immersing them completely in 4% paraformaldehyde solution for 4 hours at 4°C. It is important to keep the fixation times consistent between samples.

1.3. Cryoprotection:

- Cryoprotect spinal cords sequentially by immersing them in 15%, 20%, and 30% sucrose in 0.1M PB solutions for 24h, each at 4°C, allowing enough time for the tissue to equilibrate in each solution.

1.4. Mounting and Embedding:

- Freeze-mount spinal cords in optimal cutting temperature (OCT) compound. To ensure consistent, high-quality sections, first dry the exterior of the spinal cord sections by gently patting them with gauze or a paper towel. Align the spinal cords in a consistent orientation within the OCT-filled cryomold, making sure that the regions of interest (e.g., the middle of the lumbar enlargement) are aligned across multiple samples to be captured in the same plane during sectioning. Once the submerged tissues are properly aligned, place the cryomold on a bare metal shelf in a -80°C freezer for rapid freezing. Up to four spinal cords can be accommodated in a single 25mm x 20mm x 5mm Tissue-Tek Cryomold. Arranging the tissue as densely as possible will help conserve reagent costs.

1.5. Sectioning:

- Cut frozen sections at 14μm thickness using a cryostat, mounting each tissue embedded strip onto superfrost slides. -15-20°C is a reliable temperature for smooth cutting. As an alternative to using a roll plate, consider the use of a paintbrush to gently pull each strip of OCT embedded cords as it is sectioned; this alternative to a roll plate can help avoid creases in the delicate mounting medium and the embedded tissue due to compression from the glass. To minimize reagent costs, aim to arrange sections on slides as compactly as feasible.

1.6. Slide Preparation:

- After sectioning, allow the slides to equilibrate at room temperature for 1 hour, and then freeze them. This step helps improve the adhesion of the tissue sections to the slides. Once slides have been allowed to adhere for 1 hour, freeze the slides at -80°C until the day staining begins.

1. RNAscope + IHC Staining Day 1

2.1. Slide Preparation

- Let slides sit at room temperature for 30 minutes.
- Be sure to include a negative control slide or include a negative control section on a slide for comparison. For the negative controls, follow all instructions except do not add any fluorescent reporters to solutions (Opal Dye or Alexafluor conjugated secondary antibodies). For an RNAscope probe negative control, use provided ACDBio negative control probes which encode for bacterial transcripts.

2.2. Reagent Preparation

- Prepare the following solutions with deionized water (dH^2^O):
  - 1x RNAscope target retrieval reagent (ACDbio): 50 mL for every 5 slides, assuming 50mL conical will be used for the heat incubations (5 mL 10x target retrieval reagent + 45 mL dH^2^O)
  - 5x saline sodium citrate (SSC): 500 µL per slide (2 mL 20x SSC stock + 6 mL dH^2^O)
  - Ethanol solutions: 50%, 75%, and 100%
- Preheat a water bath to 98°C and slide oven to 67°C.

2.3. Target Retrieval

- Add 60 mL of 1x target retrieval reagent to a heat-tolerant coplin jar. Place the jar in the the water bath, keeping the water bath lid open to prevent condensation from dripping into the jar.
- Wash slides in 0.1M PBS to remove OCT (2x 10 min washes).
- Take RNAscope probes out of the fridge to warm up to room temperature during dehydration steps.
- Dehydrate slides in ethanol solutions for 5 min each at room temperature, in the following order: 50% EtOH, 75% EtOH, 100% EtOH, and again at 100%EtOH.
- Allow slides to air dry for 5 minutes at room temperature.

2.4. Hydrogen Peroxide Treatment

- Use the hydrophobic pen to draw a hydrophobic barrier around the tissue sections. Ensure that an equal size barrier is drawn for all slides in the study.
- Apply RNAscope hydrogen peroxide solution to the tissue sections, ensuring they are completely covered. Place slides in a humidity control tray with a moistened absorbent pad.
- Incubate for 10 minutes at room temperature, then remove excess liquid.

2.5. Target Retrieval (Continued)

- Carefully place slides into the coplin jar containing 1x target retrieval in the water bath. Incubate for 5 minutes.
- *Carefully* remove slides and place onto a tray for baking. For thicker (14μm sections spinal cord tissue) it is essential to bake the sections dry for 30 minutes at 67°C to avoid tissue loss. It is also recommended to gently pipette all washes from this point on.

2.6. Protease III Treatment

- Place slides in the humidity control tray and cover with RNAscope Protease III. Incubate for 10 minutes at 40°C in the oven.
- Prepare probes during this incubation (see 2.7).
- Remove slides from the oven and wash in dH^2^O for 2 x 2 minutes at room temperature.

2.7. Probe Hybridization

- Prepare RNAscope probes according to ACDbio instructions, ensuring appropriate dilutions and combinations.
- Apply a consistent amount of prepared probes to each slide, covering all sections completely (approximately 300 µL for a full slide).
- Incubate for 2 hours at 40°C in the oven.
- Gently wash slides in 1x RNAscope wash buffer on for 2 x 2 minutes at room temperature. To prevent tissue from falling off slides, only a very gentle orbital shaker is recommended for washes.

2.8. Optional Overnight Storage

- If desired, store slides flat on the incubation tray in the prepared 5x SSC overnight at 4°C. Alternatively, proceed to Day 2 of the protocol.

1. Day 2: RNAscope and Immunohistochemistry

3.1. Amplification

- Preheat the oven to 40°C.
- Remove slides from the fridge and wash in 1x wash buffer for 2 x 2 minutes at room temperature. Remove excess liquid.
- Apply AMP 1 and incubate for 30 minutes at 40°C.
- Meanwhile, allow the TSA to warm to room temperature.
- Wash slides in 1x wash buffer for 2 x 2 minutes.
- Apply AMP 2 and incubate for 30 minutes at 40°C.
- Wash slides in 1x wash buffer for 2 x 2 minutes.
- Apply AMP 3 and incubate for 15 minutes at 40°C.
- Wash slides in 1x wash buffer for 2 x 2 minutes.

3.2. Signal Development

Perform the following steps for all applicable channels (C1, C2, and C3, beginning with C1, and then repeating the process for C2 and then C3)

- Apply HRP-Cx to open the Cx probes. Incubate in the humidity control tray in the oven for 15 minutes at 40°C.
- Meanwhile, during the HRP-Cx incubation, prepare the Cx dye solution (~300 µL per slide) by diluting Opal dyes in TSA (ACDbio) at a 1:250 concentration.
- Remove slides from the oven after HRP-Cx incubation is complete.
- Wash in 1x wash buffer for 2 x 2 minutes.
- Apply 250 µL of Cx dye solution to each slide.
- Incubate for 30 minutes at 40°C.
- Wash slides in 1x wash buffer for 2 x 2 minutes.
- Apply HRP-Blocker to close the Cx probes. Incubate for 15 minutes at 40°C.
- Wash slides in 1x wash buffer for 2 x 2 minutes

3.3. Primary Antibodies

- During the final HRP-Blocker step, prepare the primary antibody solution, diluting the antibody in co-detection diluent (CDD) from ACDbio. Use approximately 300 µL per slide.
- Incubate slides overnight at 4°C without agitation, ensuring the slides will not be disturbed.

1. Day 3: RNAscope and Immunohistochemistry

4.1. Secondary Antibodies

- Wash slides with PBS for 2 x 5-minute washes.
- Prepare secondary antibodies in CDD.
- Incubate slides for 2 hours at room temperature.
- Wash slides in distilled water for 2 x 5-minute washes.

4.2. DAPI Counterstaining (optional) and Coverslipping

- Drop DAPI solution over the slide, ensuring sections are covered.
- Incubate for 30 seconds at room temperature, then immediately pipette off the liquid.
- Immediately cover with Prolong mounting medium and coverslip.
- Place slides in a dry, light protected environment to dry overnight at room temperature.

4.3. Slide Storage and Imaging

- Store slides at room temperature until imaging.
