# Supplementary figures and images for: Combining RNAscope and immunohistochemistry to visualize inflammatory gene products in neurons and microglia
Source: Front Mol Neurosci. 2023 Aug 17;16:1225847. doi: 10.3389/fnmol.2023.1225847 (PMC10470653; doi:10.3389/fnmol.2023.1225847)

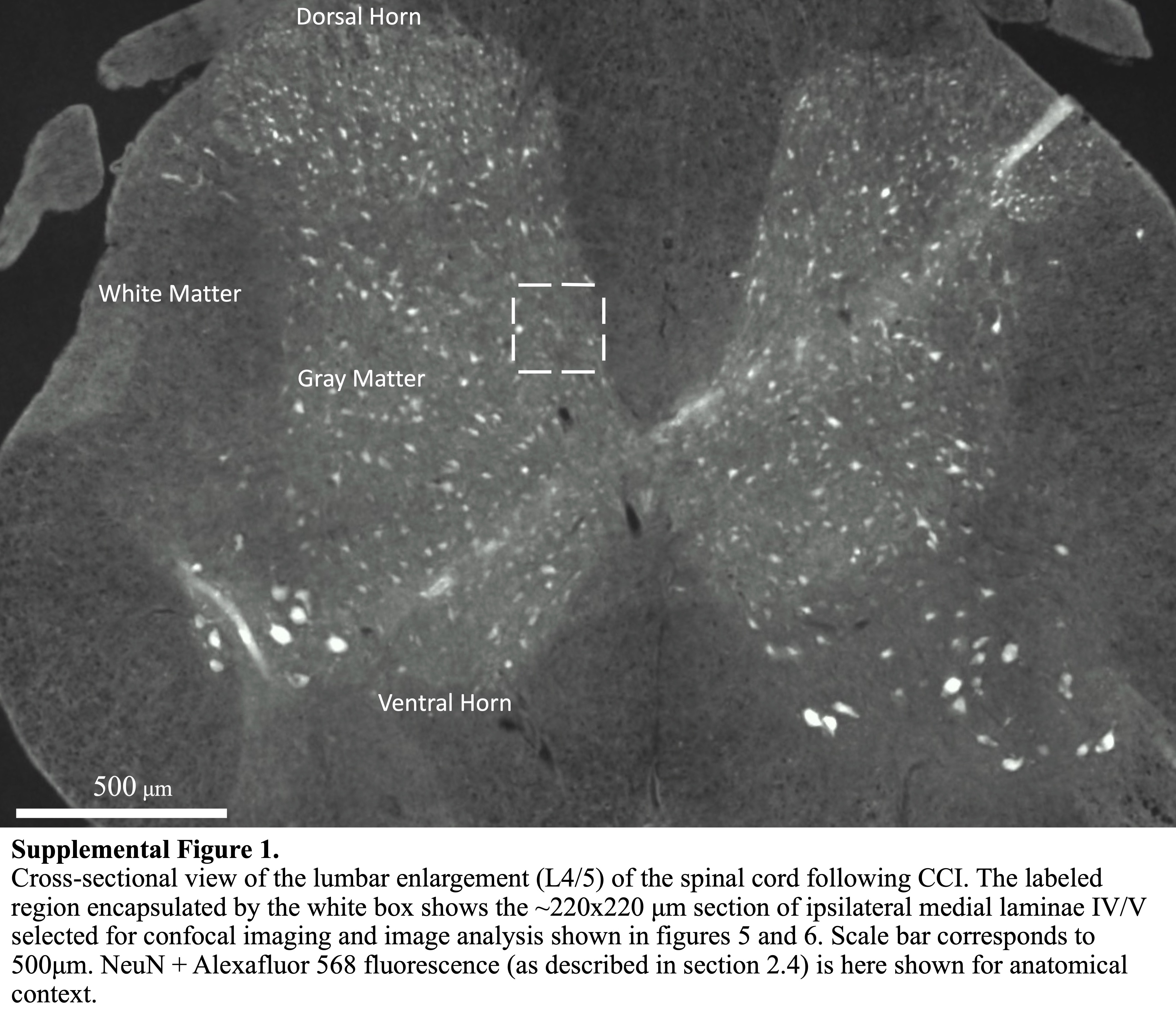

Supplement: Supplementary file 2 [file Image_1.jpg]
